# The impact of COVID-19 restrictions on road traffic and noise pollution of the urban street, a case study from Krakow, Poland

**DOI:** 10.1007/s11356-024-35365-5

**Published:** 2024-10-26

**Authors:** Karol Plesiński, Mariusz Cholewa

**Affiliations:** Faculty of Environmental Engineering and Geodesy, Department of Hydraulic Engineering and Geotechnics, University of Agriculture in Krakow, Mickiewicza Street 24/28, 30-059 Kraków, Poland

**Keywords:** COVID-19 pandemic, COVID-19 restrictions, SARS-CoV-2 coronavirus, Noise pollution, Street traffic, Acoustic parameters, Main urban street

## Abstract

The study determined the impact of restrictions introduced by the Ministry of Health in connection with the COVID-19 pandemic on traffic and sound pressure levels in the university building and its immediate vicinity. Mickiewicza Street was selected for the study. It is one of the city’s busiest streets, located relatively close to the old historic center, and is the main artery connecting the north and south of the city. There are residential buildings on this street, but primarily offices and institutions, as well as schools and universities. Noise near the street and in buildings located relatively close to the road can be a serious problem in places where peace is required (e.g., libraries, reading rooms, university halls). Therefore, the acoustic comfort needed in these places may be disturbed, and the perception of knowledge acquisition may be limited. The aim of the work was achieved through measurements and analysis of street traffic intensity and sound pressure levels, taking into account pandemic restrictions. Then, statistical analyses were performed, which showed significant variability in traffic intensity and sound pressure values in individual periods of the pandemic. The pandemic’s beginning was mainly characterized by a significant decrease in the above data, deviating from the norm. In the pre-pandemic period, an average of 47,733 vehicles passed through the street daily, and the median sound pressure was *L*_eq.OUT_ = 62.0 dBA outside the building and *L*_eq.INS_ = 44.0 dBA inside. During the greatest restrictions, a period called “lockdown,” the number of cars driving decreased a little to an average of 44,153 per day, but there were days with 10,000–20,000 cars passing. In turn, noise decreased to 59.9 dBA for *L*_eq.OUT_ and 43.2 dBA for *L*_eq.INS_. Between the first and second waves of infections, traffic was comparable to the post-pandemic period (an average of 69,027 cars per day), and noise also increased to *L*_eq.OUT_ = 64–65 dBA and close to *L*_eq.INS_ = 47.5 dBA. Studies have shown that noise on Mickiewicza Street is mainly caused by traffic (correlation coefficient: *r* = 0.92 for* L*_eq.OUT_ and *r* = 0.86 for *L*_eq.INS_). The sound pressure level is high outside (*L*_eq.OUT_ = 63.9 dBA) but relatively low inside the building (*L*_eq.INS_ = 47.0 dBA) and should not negatively affect university staff and students (*L*_eq.WH_ < *L*_WH.acc_). Based on the analyses, three stages show society’s approach to the COVID-19 pandemic: (1) fear of the COVID-19 pandemic; (2) relaxation after restrictions; (3) getting used to the COVID-19 pandemic. These periods did not correlate with the development of the pandemic or the number of people infected and dead.

## Introduction

Sound is ubiquitous in everyday life. The expression noise is a sound that is a particle of energy. Sound, due to i.a. its volume, frequency, impulsiveness, and tonality, may turn into noise and become a nuisance to people and the environment (ISO 1996–1:^2016^), having a harmful physiological and psychological impact on people (Alvares-Sanches et al. 2023; Ayush et al. 2022; Mamun et al. 2017; Stansfeld and Matheson 2003). Harmful or annoying noise levels cause noise pollution.

Noise in the environment is one of the most significant ecological pollutants (Jóźwiak 2014; Tandel and Macwan 2017). Its leading cause is the progressive urbanization of cities and the proximity of housing estates and office buildings to communication roads, which poses a noise threat to people staying there (Poniatowski 2022). In highly developed countries, the sound pressure level is often so high that it negatively affects the utility value of areas and buildings (Rącka and Szopińska 2017). It is also considered the third most dangerous environmental pollution and human health (Bouzir et al. 2017; Freitas et al. 2018). Subjective and objective research on the impact of noise on human well-being, activity, and health has been conducted for many years (Amoatey et al. 2023; Casla-Herguedas et al. 2023; Khomenko et al. 2022; Ohaeri and Obafemi 2024). In Poland in the 1990s, 21% of the area and 33% of the population were exposed to excessive noise (Maranda 2008). This trend is increasing mainly due to the expansion of road infrastructure (Szopińska 2015; Singh et al. 2023), as well as the increase in permissible values (Table 1) (Regulation 1980, 1998, 2004, 2007, 2012; Announcement 2014).

**Table 1:** Permissible noise levels in the environment caused by road noise (short-term indicators) (according to the Regulation 1980, 1998, 2004, 2007, 2012; Announcement 2014)

Years	Type of development									
	1			2				3*		
	Residential areas with a small number of shops and service outlets, located near streets with traffic of up to 1000 vehicles per hour			Residential areas with a small number of shops and service outlets, located near streets with traffic of up to 2000 vehicles per hour				Central parts of cities with residential buildings or streets with traffic intensity exceeding 2000 vehicles per hour		
	*L*_eq.DAY_	*L*_eq.NIGHT_	*L*_eq.SHORT_	*L*_eq.DAY_	*L*_eq.NIGHT_	*L*_eq.SHORT_	*L*_eq.DAY_		*L*_eq.NIGHT_	*L*_eq.SHORT_
1980	50	40	75	55	45	80	60		50	85
	Areas of single-family housing development			Areas of multi-family housing development and collective housing				Areas in the inner-city zone of cities with over 100,000 inhabitants with compact housing development		
	*L*_eq.DAY_	*L*_eq.NIGHT_	-	*L*_eq.DAY_	*L*_eq.NIGHT_	-	*L*_eq.DAY_		*L*_eq.NIGHT_	-
1998	55	45		60	55		65		55	
2004, 2007	55	50		60	50		65		55	
2012, 2014	61	56		65	56		68		60	

Where: *L*_eq.SHORT_ — equivalent sound pressure level short-term

^*^Column no. 3 referring to the university building

Road noise varies over time and emitted by vehicles passing at different frequencies and intensities. It is usually a superposition of the acoustic background and noise resulting from vehicle movement, which includes the noise generated at the tire-road interface and the noise of the vehicle’s drive unit (Gierasimiuk and Motylewicz 2014). Road noise pollution depends mainly on the number of vehicles and their types (Ranpise and Tandel 2022), as well as on other factors such as condition and type of road surface (Licitra et al. 2017), traffic jams (Akhtar et al. 2012; Tandel and Macwan 2013), vehicle condition (Kumar et al. 2014; Staiano 2018), and speed of driving vehicles (Abbaspour et al. 2006; Tian et al. 2014).

In Poland, road noise standards were significantly increased (and therefore relaxed) in 2012 (Table 1). Initially (in 2007), they were as follows: for areas with type 1 development — *L*_eq.DAY_ and *L*_eq.NIGHT_: 55 and 50 dB (increase by 6 dB); for areas with type 2 development — *L*_eq.DAY_ and *L*_eq.NIGHT_: 60 and 50 dB (increase by 5 and 6 dB, respectively); for areas with type 3 development — *L*_eq.DAY_ and *L*_eq.NIGHT_: 65 and 55 dB (increase by 3 and 5 dB, respectively). It should be noted that an increase in the noise level by 3 dB translates into a doubling of the sound pressure and by 10 dB — into a tenfold increase in this pressure (Draft Regulation 2012a). This shows the practical scale of the rise in standards made in 2012 (e.g., increasing the permissible noise level from 55 to 61 dB is not an increase “only by 11%” but means as much as a fourfold increase in the permissible acoustic power) (Poniatowski 2022). Proposals for changes to the regulation on permissible noise levels in the environment were mainly justified by economic reasons, such as the need to use expensive acoustic protection during road investments (Draft Regulation 2012a). The project was subject to extensive public consultations, during which many negative opinions were submitted (Draft Regulation 2012b). Significantly, almost all views of entities dealing with environmental protection and acoustics did not support raising the maximum permissible noise values. In contrast, opinions approving the increasing noise standards (or no comments) came mainly from entities interested in raising these standard values or people unrelated to noise issues.

Based on acoustic maps prepared under the first stage of Directive 2002/49/EC, it was found that approximately 3.8 million inhabitants of 12 large agglomerations in Poland are exposed to road noise exceeding 55 dB during the day, and 2.8 million 50 dB during the night. According to the same directive, no resident of the European Union should be exposed to noise that endangers health or quality of life (Directive 2002/49/EC 2002). As many as 16% of the European population living in agglomerations with over 250,000 inhabitants are exposed to harmful levels of road noise. For comparison, 1.0% of the population are exposed to rail noise. In contrast, only 0.1% are exposed to aircraft and industrial noise (EEA Report 2014).

The effects of exposure to noise on human health are hazardous. Medical research conducted over many years has shown that noise causes hearing impairment or permanent damage, damage to the nervous system and psyche (for example, fatigue, drowsiness or irritability, prolonged reaction time, decreased concentration, anxiety, insomnia, aggression, and stress, and the resulting consequences for the entire body and psyche) and damage to internal organs (for example, cardiovascular, digestive, and musculoskeletal systems, weakening of the immune system, the so-called post-noise syndrome) (Berglund et al. 1999; Tobías et al. 2015; Singh et al. 2018). Noise affecting people at night is particularly harmful (Münzel et al. 2020). Long-term exposure to environmental noise, including road noise, causes 12,000 premature deaths in Europe each year and contributes to 48,000 new cases of ischemic heart disease (EEA Report 2014).

The COVID-19 pandemic, and especially the restrictions introduced by governments around the world, have resulted in reduced mobility (especially in the first phase of the pandemic), which has usually translated into a reduction in road noise (Abdmouleh and Dahech 2024; Caraka et al. 2021; Trudeau et al. 2023; Vílchez-Gómez et al. 2023), aviation noise (Amoatey et al. 2022; Audrin et al. 2022; Zijadić 2021), and even maritime noise (Čurović et al. 2021; Mrčelić et al. 2023).

The COVID-19 pandemic resulted in a significant reduction in environmental pollution, including noise pollution. Notably, biological systems were partially restored (Zambon et al. 2021). The reduction in noise pollution was due to decreased sound pressure levels and spectral changes. This suggests a shift from anthropogenic to animal sources in the acoustic environment (Vida Manzano et al. 2021). Spennemann and Parker (2020) also noted that during the COVID-19 pandemic, there was a significant but temporary change in urban soundscapes. They also point out that the “sounds of the city” are a cultural heritage that should be protected. Mimani and Singh (2021) also observed changes in noise pollution during the COVID-19 pandemic. They showed the most significant decrease in sound pressure levels in the shopping zone, which was associated with reduced shop activities. A relatively small decrease was observed in the mixed zone. Despite the restrictions, people performed their daily duties, such as shopping and work (not all industries were closed). However, no changes were noticed in parks, which may indicate small anthropogenic sound sources.

Despite the reduction in noise pollution during the COVID-19 pandemic (Alsina-Pagès et al. 2021; Vida Manzano et al. 2021), people potentially exposed to noise pollution often perceive noise subjectively, which does not always correlate with objective field measurements of sound pressure (Choi et al. 2021; Yildirim et al. 2023). During the pandemic, there has been a general increase in reports of noise pollution, especially in residential buildings and industrial plants. Since the state of emergency was declared, most residents have been working from home, so noise nuisance from residential areas is greater. Furthermore, with less road traffic (Casla-Herguedas et al. 2023; Choi et al. 2021), a general reduction in noise levels can be expected, making other sources of noise potentially more bothersome, as individual noise sensitivity has increased (Zambon et al. 2021). Only Maggi et al. (2021) showed the opposite trend, suggesting increased perceptions of calmness and happiness among those exposed to less mechanical noise and increased biological sounds.

The study determined the impact of restrictions introduced by the Ministry of Health in connection with the COVID-19 pandemic on street traffic and street noise pollution in the immediate vicinity and the building of the University of Agriculture in Krakow. Acoustic conditions that may constitute noise pollution in the area of the road were determined, affecting the quality of work performed by employees of the University of Agricultural in Krakow. The goal was achieved by measuring the sound pressure level in one of the university rooms and outside the building from Mickiewicza Street and the street traffic intensity.

## Methodology

### Study area

**Fig. 1:**
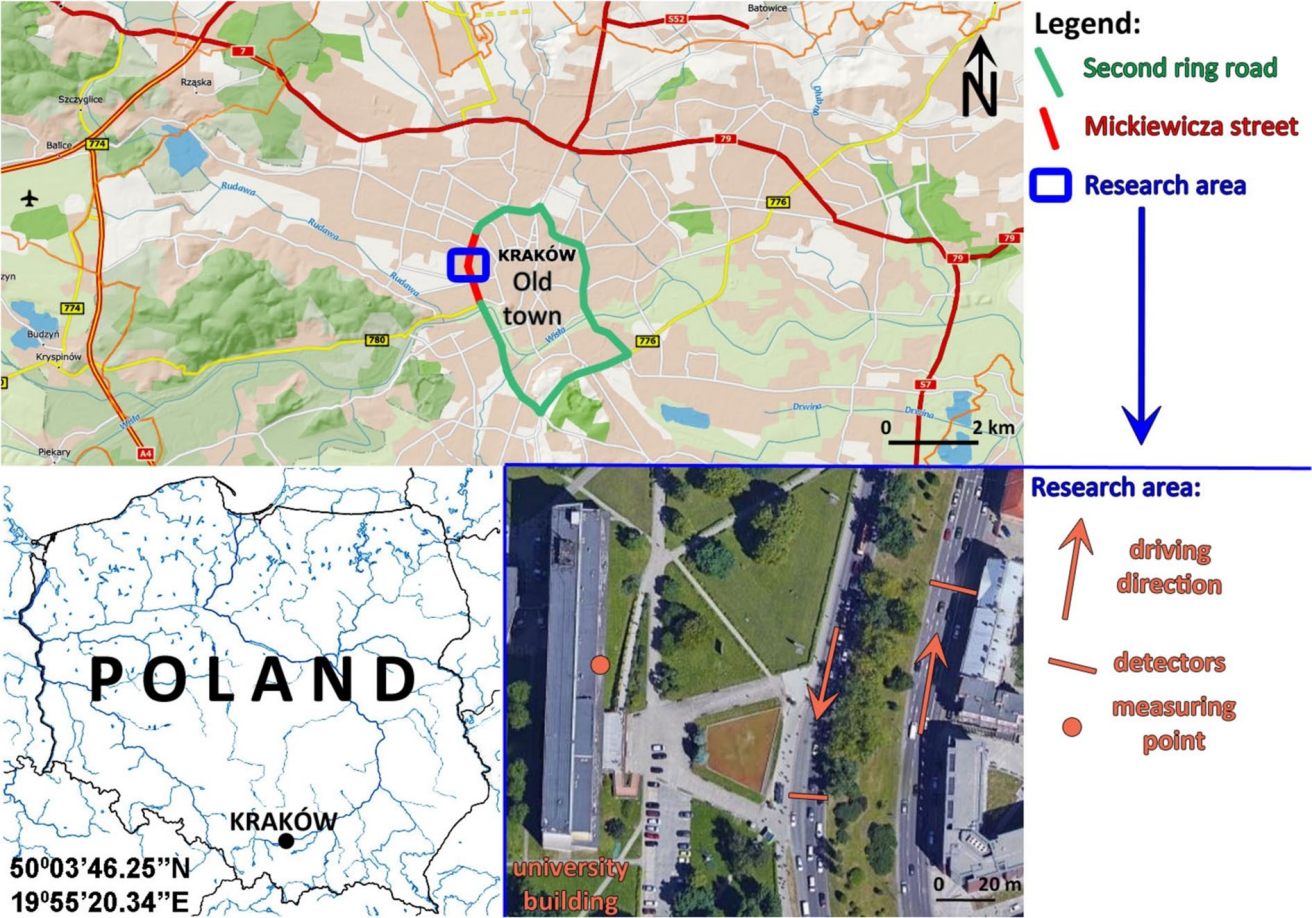
Location of the research object

The sound pressure level was measured in the building of the University of Agriculture in Krakow and its vicinity outside, near Miskiewicza Street. It runs from south to north and is one of the roads forming the second ring road of Krakow, part of the so-called Avenue of the Three Prophets (Fig. 1).

Avenue of the Three Prophets has a rich history closely related to Krakow’s nineteenth- and twentieth-century development. The creation of this communication artery resulted from the need to organize and facilitate road traffic around the rapidly developing city, which at that time was beginning to expand its borders beyond the historic center. Their history also fits into the broader context of the modernization of the urban infrastructure of Krakow, which underwent significant changes during that period. A significant part of the areas where the current avenues run was outside the city walls and along the former fortifications and defensive ramparts, which began to lose their importance. At that time, a plan was drawn up to build a wide communication artery to connect the developing suburbs with the center of Krakow, creating a ring road around the most important districts of the city.

The design of these avenues was linked to the growing role of road and urban infrastructure, which was to serve not only as a road for cars but also as an elegant promenade and a representative street of the city.

Currently, these avenues are one of the busiest roads in the city, located relatively close to the old, historic city center (Supranowicz 1995). Due to their importance in location and communication, Avenue of the Three Prophets is a key element of street traffic in Krakow. Numerous bus lines run along this route, allowing residents and tourists to move quickly.

One of the main problems of Avenue of the Three Prophets is traffic congestion. Thousands of cars pass through them daily, leading to traffic jams, especially during rush hours. Additionally, the proximity of historic districts means that widening the streets or building new road connections is very limited.

City plans aim to reduce car traffic on Avenue of the Three Prophets by promoting public transport and ecological alternatives, such as bicycles or the development of car-sharing. There is also an increasing emphasis on improving air quality and reducing exhaust emissions in the city center (plans to introduce a Clean Transport Zone), which is becoming a priority due to air pollution. Therefore, Avenue of the Three Prophets, although necessary for road traffic in Kraków, also poses a challenge from the ecology and urban infrastructure perspective.

Residential buildings, but above all, offices and institutions, as well as schools and universities (including University of Science and Technology /abbreviation: AGH/, University of Agriculture /abbreviation: UR/, Polish Academy of Science), are located on this street. Noise pollution near the street and in buildings located relatively close to the road may be a severe problem in places where quiet is required (e.g., libraries, reading rooms, university lecture halls). Therefore, the needed acoustic comfort in these places may be disturbed, and the perception of knowledge acquisition may be limited.

### Street traffic data

The Municipality Road Administration of Krakow made street traffic measurements available. This institution measures traffic flow on each lane separately. To the south, the detectors were located opposite the university building, next to the “AGH/UR” bus shelter. To the north, the detectors were located opposite the building at Mickiewicza St., 15, opposite the university building (Fig. 1). Traffic intensity data was made available at a time interval of 15 min.

### Meteorological data

Meteorological measurements were made available by the Institute of Meteorology and Water Management. They contain data on precipitation per day, air humidity, air temperature, wind speed, and direction. All data was made available at a time interval of 15 min.

### Sound pressure level measurements

Sound pressure level measurements were taken in the area of Mickiewicza Street. The decibel meter was located 75 m and 105 m from the nearest road lanes running south and north, respectively (Fig. 1), in front of the building of the University of Agriculture in Krakow (Mickiewicza St., 24/28) and one of the rooms of this building. Measurements were performed 24/7, non-stop. The choice of measurement periods was mainly determined by the restrictions introduced by the Ministry of Health in connection with the COVID-19 coronavirus pandemic prevailing at that time.

The number of COVID-19 cases and deaths was not provided because the Ministry of Health released this data with a 1-day delay. Only the trend in incidence was taken into account. The measurements were made with Voltcraft SL-451 decibel meters, which comply with the IEC 61672–1 standard (2013). The following technical parameters characterize the meter: measurement range from 30 to 130 dB, at a frequency of 31.5–8 Hz; averaging time constant Fast (25 ms) —recommended for noise measurements that are characterized by rapid variability (Kraszewski et al. 1996; Regulation 2011); measurement accuracy ± 0.1 dB; A frequency filter weighting; measurement time: 24 h; measurement time interval: 1 s. Both decibel meters were connected to a laptop, where the measured parameters were recorded using the Voltsoft Client 1.98 program. The room was located on the side of the analyzed street. Outsiders were prohibited from entering or staying in the room during the measurements.

### Sound index calculations

**Fig. 2:**
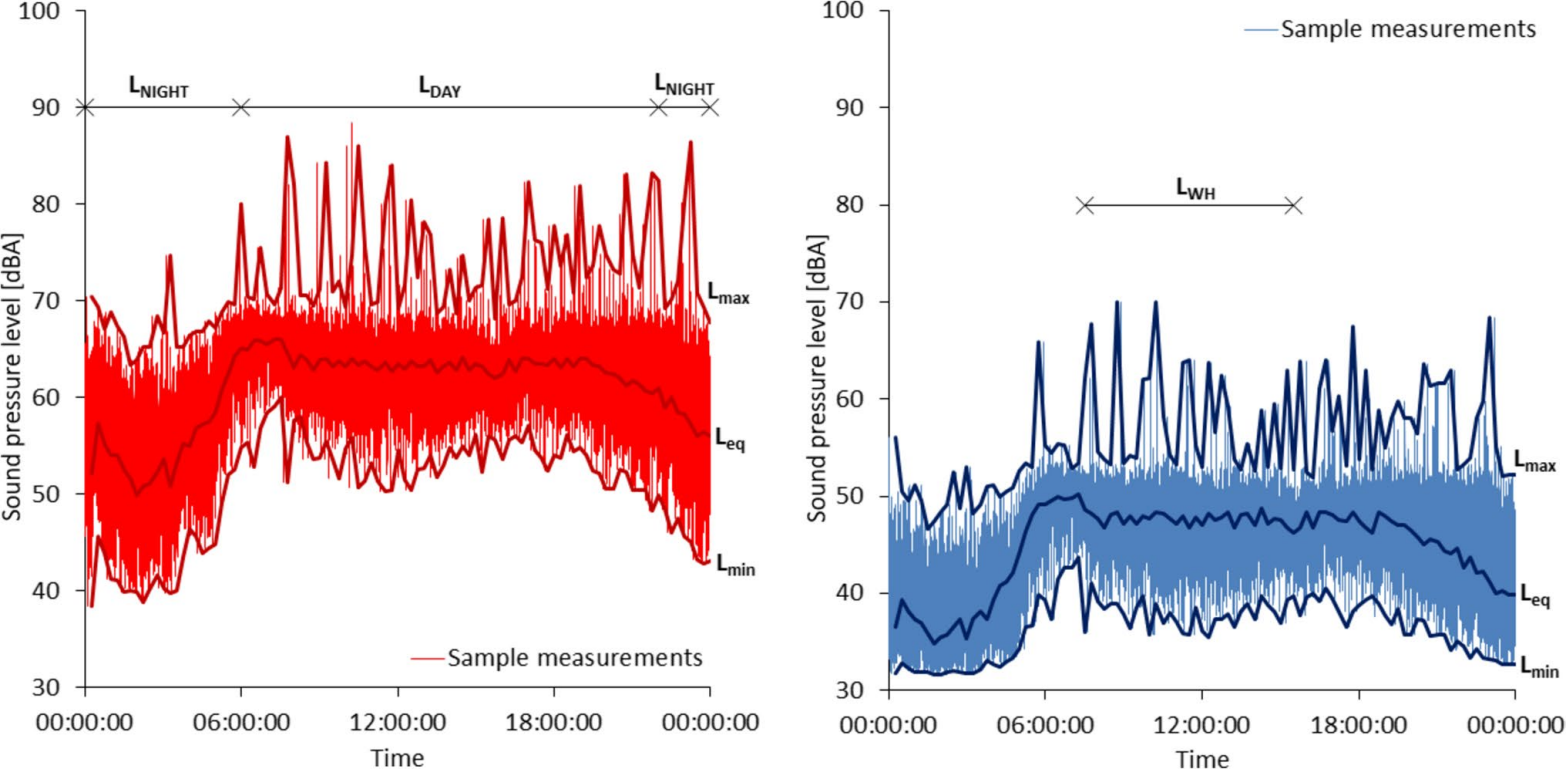
Example of raw sound pressure level measurements outside the building (left) and inside (right) considering acoustic parameters

Sound indices were calculated for every measured period (Fig. 2):

- ${L}_{\text{eq}.\text{OUT}}$ — equivalent sound pressure level measured at an interval of 15 min outside the building,
- ${L}_{\text{min}.\text{OUT}}$ — minimum sound pressure level measured at an interval of 15 min outside the building,
- ${L}_{\text{max}.\text{OUT}}$ — maximum sound pressure level measured at an interval of 15 min outside the building,
- ${L}_{\text{eq}.\text{INS}}$ — equivalent sound pressure level measured at an interval of 15 min inside the building,
- ${L}_{\text{min}.\text{INS}}$ — minimum sound pressure level measured at an interval of 15 min inside the building,
- ${L}_{\text{max}.\text{INS}}$ — maximum sound pressure level measured at an interval of 15 min inside the building,
- ${L}_{\text{eq}.\text{DAY}}$ — the equivalent sound pressure level measured at an interval of 15 min outside the building during the day (from 6:00 a.m. to 10:00 p.m.),
- ${L}_{\text{max}.\text{DAY}}$ — maximum sound pressure level measured at an interval of 15 min outside the building during the day (from 6:00 a.m. to 10:00 p.m.),
- ${L}_{\text{DAY}.\text{acc}}$ — maximum permissible sound pressure level during the day (from 6:00 a.m. to 10:00 p.m.) according to Announcement (2014),
- ${L}_{\text{eq}.\text{NIGHT}}$ — equivalent sound pressure level measured at an interval of 15 min inside the building during the night (from 10:00 p.m. to 6:00 a.m.),
- ${L}_{\text{max}.\text{NIGHT}}$ — maximum sound pressure level measured at an interval of 15 min inside the building during the night (from 10:00 p.m. to 6:00 a.m.),
- ${L}_{\text{NIGHT}.\text{acc}}$ — maximum permissible sound pressure level during the night (from 10:00 p.m. to 6:00 a.m.) according to Announcement (2014),
- ${L}_{\text{eq}.\text{WH}}$ — equivalent sound pressure level measured at an interval of 15 min outside the building during the work hours (from 7:30 a.m. to 3:30 p.m.),
- ${L}_{\text{max}.\text{WH}}$ — maximum sound pressure level measured at an interval of 15 min outside the building during the work hours (from 7:30 a.m. to 3:30 p.m.),
- ${L}_{\text{WH}.\text{acc}}$ — maximum permissible sound pressure level during the work hours (from 7:30 a.m. to 3:30 p.m.) according to Boczkowska et al. (2014).

### Data on restrictions caused by the COVID-19 pandemic

**Fig. 3:**
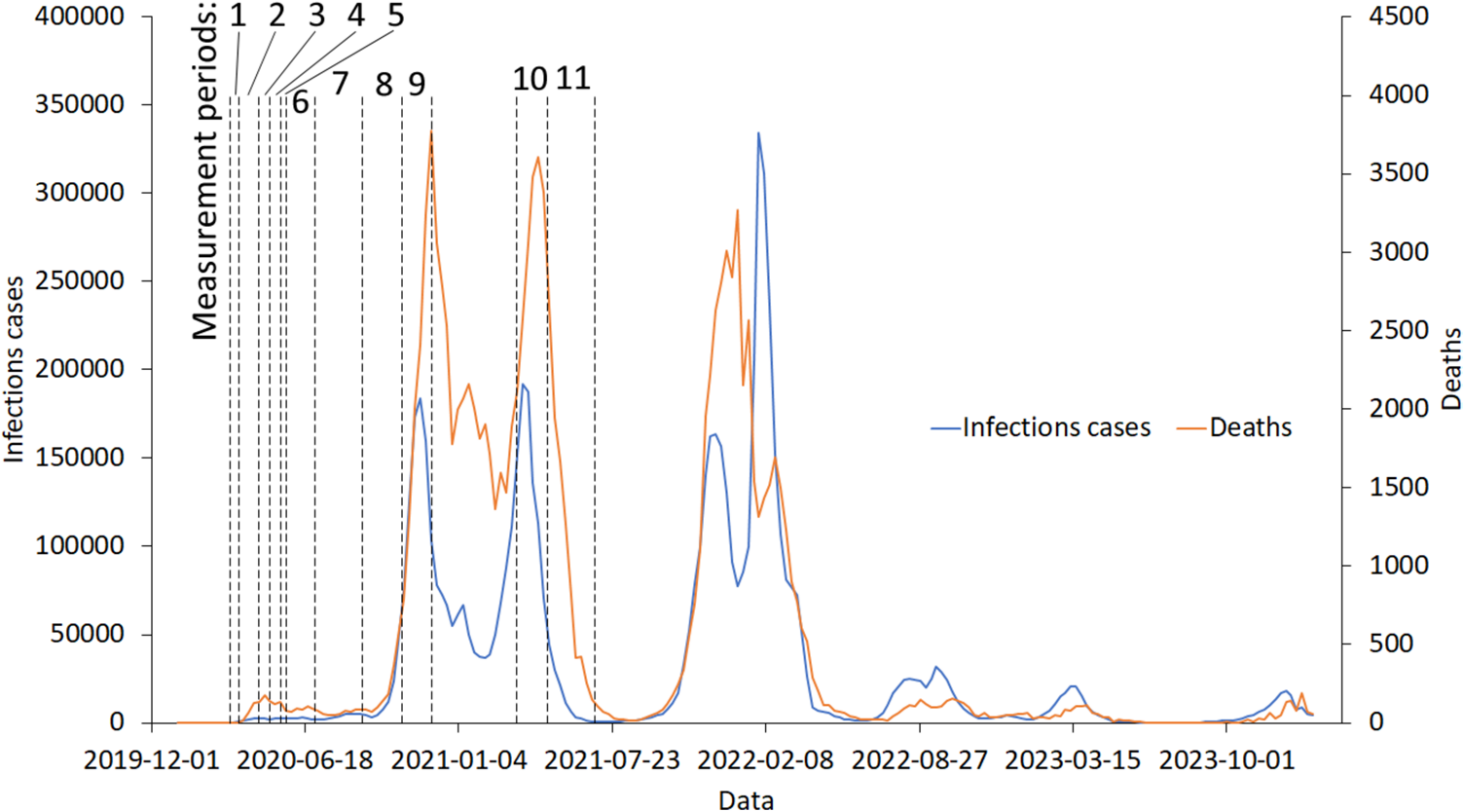
Number of illnesses and deaths caused by the COVID-19 pandemic, including measurement periods

The measurement period was divided into shorter sections, which depended on the restrictions introduced by the Ministry of Health. Their introduction most often resulted from the development of the COVID-19 pandemic and, more precisely, from the number of infections and deaths (Duszyński et al. 2021; Ministry of Health 2024). Therefore, 11 measurement periods were distinguished (Fig. 3):

- Period 1, “pre-lockdown,” from 12 to 24 March 2020 (13 days)—the functioning of the education system units was suspended (classes at universities, schools, kindergartens, and nurseries were suspended). Restrictions were also introduced on catering and entertainment activities and the operation of shopping malls. An epidemiological threat has been introduced. The number of infections was 264 people per week. Number of deaths: 5 for the entire period.
- Period 2, “lockdown,” from 25 March to 19 April 2020 (36 days)—on 25 March, movement restrictions were introduced. It was impossible to move except for living, health, and professional purposes. Public transport could accommodate half the number of people seated in a given vehicle. A total ban on gatherings (except for close relatives) has been introduced. On 1 April, further restrictions on the movement of people were introduced. People under 18 years of age could only go outside under the supervision of an adult. Parks and boulevards were closed. Beauty salons, hairdressers, and tattoo studios were closed. There were limitations in stores: three people per one checkout position. Hardware stores were closed on weekends. It was a period called lockdown. The number of infections was 2052 people per week. Number of deaths: 85 people per week.
- Period 3, “1st stage,” from 20 April to 3 May 2020 (14 days)—the first stage of lifting restrictions. They were reducing the limits of people in shops and places of worship. Forests and parks were opened. Youths over 13 will be able to go outside without adult supervision. Number of infections: 2316 people per week. Number of deaths: 158 people per week.
- Period 4, “2nd stage,” from 4 to 17 May 2020 (14 days)—the second stage of lifting restrictions. Sports fields were opened, and amateur sports were allowed to be practiced in open spaces. Shopping malls and DIY stores were also opened on weekends, and hotels resumed operations. Fitness clubs, swimming pools, gyms, and recreational spaces (e.g., playgrounds) are still closed. Libraries, museums, and facilities related to cultural activities were opened. Nurseries and kindergartens were opened on 6 May 2020. Number of infections: 2441 people per week. Number of deaths: 127 people per week.
- Period 5, “3rd stage,” from 18 to 25 May 2020 (8 days)—the third stage of lifting restrictions. Hairdressing and beauty salons were opened. Food outlets were also opened but with certain limitations. Youths up to 13 years of age could go outside without an adult. The limit of passengers in public transport has been increased (one person per one seat). Sports facilities were opened. Number of infections: 2674 people per week. Number of deaths: 78 people per week.
- Period 6, “4th stage,” from 26 May to 1 July 2020 (37 days) — the fourth stage of lifting restrictions. It was possible to conduct practical classes in post-secondary schools and classes in grades 1–3 of primary schools. Possibility to consult teachers at schools for high school graduates and 8th-grade (last) pupils. It has been possible to conduct classes at universities for students in their final years of studies and classes that cannot be conducted remotely. Limits on people in shops, restaurants, hotels, and religious buildings have been abolished. Playgrounds and outdoor gyms have been opened. Number of infections: 2557 people per week. Number of deaths: 89 people per week.
- Period 7, “holiday,” from 1 July to 31 August 2020 (62 days) — no restrictions on movement. Number of infections: 3614 people per week. Number of deaths: 66 people per week.
- Period 8, “pre-2nd wave,” from 1 September to 23 October 2020 (54 days) —the beginning days of the new school and academic year. There are no restrictions on movement. Number of infections: 4030 people per week. Number of deaths: 83 people per week.
- Period 9, “2nd wave,” from 24 October to 30 November 2020 (38 days) — 2nd wave of pandemic COVID-19. Primary, secondary, and higher schools are taught remotely. Cultural institutions were closed. The limit of people staying in churches and shops has been reduced. The operations of shopping malls have been limited. Hotels could only accept business travellers. The number of infections and deaths increased significantly: 48,067 and 558 people per week, respectively.
- Period 10, “3rd wave,” from 20 March to 30 April 2021 (42 days) — 3rd wave of pandemic COVID-19. Suspension of operations of hotels, shopping malls, cultural institutions, cinemas, swimming pools, ski slopes, fitness clubs and gyms, and sports facilities. Introduction of distance learning for all types of schools. Number of infections: 123,948 people per week. Number of deaths: 3126 people per week.
- Period 11 “post-pandemic,” from 1 to 30 June 2021 (30 days) —after the wave. No restrictions. Number of infections: 1899 people per week. Number of deaths: 310 people per week.

In subsequent waves, the Minister of Health did not introduce restrictions, so measurements were not continued.

### Statistical analysis

Statistical analysis was performed for acoustic and traffic intensity data. Statistical analysis determined whether the number of passing vehicles and the variable sound pressure values in the individual periods of introduced restrictions were statistically significant. The sample was initially tested with the Shapiro–Wilk test (Shapiro and Wilk 1965) to determine the normality of the distribution. If at least one of the analyzed distributions was not normal, then the nonparametric Kruskal–Wallis ANOVA was performed (Kruskal 1952; Kruskal and Wallis 1952). This test is used to verify the hypothesis about the lack of shift in the compared distributions, i.e., most often, the insignificance of differences between the medians of the variable under study in several populations, assuming that the distributions of the variable are close. If the distribution was normal, then a one-dimensional ANOVA (Ewens and Grant 2001) was performed (which applies to the analysis of the influence of one factor on the dependent variable under study), previously checking the homogeneity of variances with the Brown-Forsythe test (Brown and Forsythe 1974). The analysis determines the absolute deviation of measurement results from the median in each studied group. This absolute deviation constitutes data subjected to precisely the same procedure performed for the analysis of variance for independent groups. Suppose the analysis of variance shows significant differences between the groups under consideration. In that case, further tests should be performed to answer the question of which of the compared populations is responsible for rejecting the null hypothesis. For this purpose, we use special post hoc tests, also called multiple comparison tests. These tests are called homogeneous grouping tests because we can obtain groups of means after applying them. This means that belonging to the same group will not differ significantly while belonging to different groups will vary significantly. Therefore, the LSD test was performed to check the dependence of individual samples, which is used for simple and complex comparisons of equal and different groups when the variances do not differ significantly (Fisher 1935; Dodge 2008). A correlation was also made between sound pressure and traffic intensity, allowing a regression formula to be developed.

In addition, a correlation was made between “meteorological conditions—acoustic indicator values” and “number of infections and deaths—traffic intensity.” In the first case, the analysis aimed to determine the impact of meteorological conditions on sound pressure levels expressed by acoustic indicators. In the second case, the analysis aimed to determine the impact of the development of the COVID-19 pandemic (morbidity and mortality caused by the SARC-CoV-2 virus) on the intensity of street traffic.

## Results

**Fig. 4:**
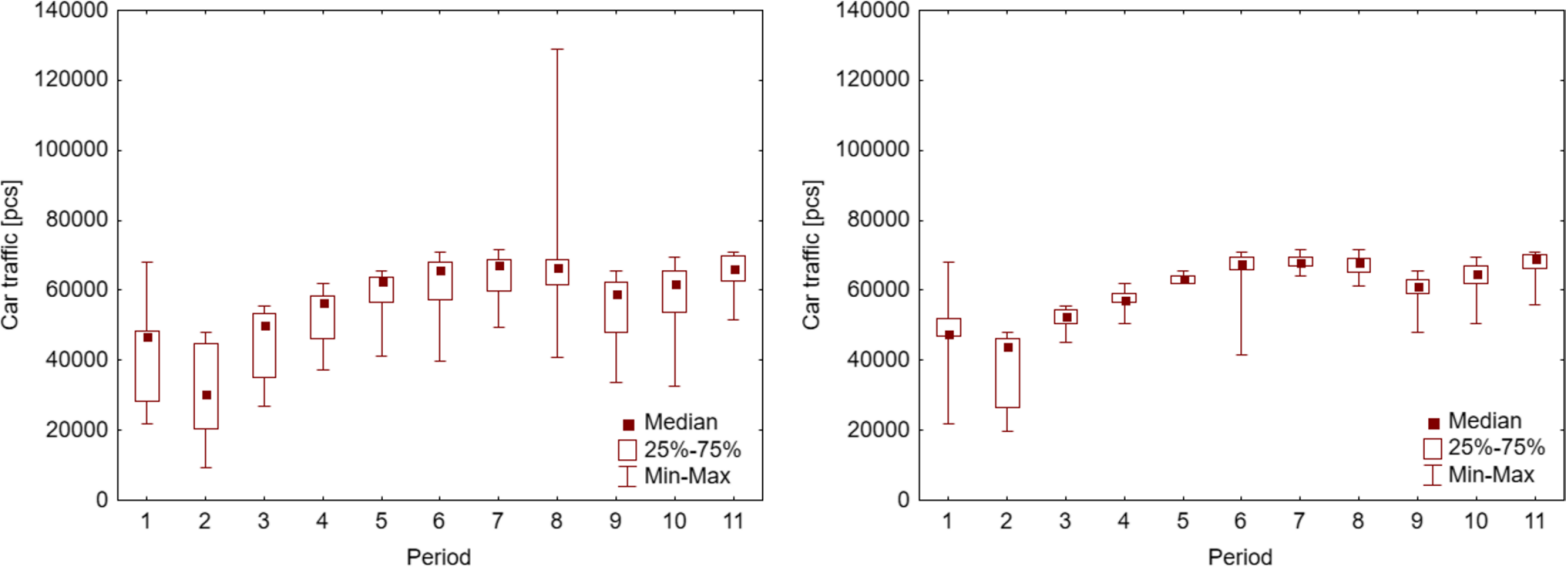
Street traffic around Mickiewicza Street in Krakow, including weekends, holidays, and special days (left) and excluding weekends, holidays, and special days (right) during individual periods of restrictions

The observation period was divided into 11 sections, which depended on the prevailing epidemiological situation in Poland and the restrictions introduced by the Ministry of Health. Figure 4 shows the distribution of the number of cars travelled in each analyzed period.

Figure 4 shows a large dispersion in the number of vehicles travelled—especially the lower limit of the range that goes well beyond the 25% quartile, which is related to the measurement of road traffic on weekends and non-working holidays. Therefore, to have a more reliable picture, it was decided to remove from the analysis the traffic measured on weekends (Saturday and Sunday), non-working holidays, long weekends, and “special” days, whose outlier data were removed. For example, the “special” days included 2 September 2020, during which children returned to school after the holidays—the number of cars travelled was 128,850 per day, almost twice the median value for this period. This step was decided after conducting a nonparametric analysis of variance, which indicated that the number of cars driving on weekends was statistically different than on working days of the week (*p* < 0.05). Hence, Fig. 4 right shows the number of cars that drove along Mickiewicza Street only on weekdays.

In the “pre-pandemic period,” an average of 22,006 to 68,007 cars passed along the street daily, with a median of 47,733. Despite the introduction of the first restrictions related to the closure of educational institutions, traffic was relatively high but decreased daily. At the beginning of the period, on 12–13 March 2020, it fluctuated at 60,000–70,000 units per day, a value similar to the last period 11 “post-pandemic.” In the first week after the introduction of restrictions (16–20 March 2020), street traffic fluctuated by around 50,000 vehicles per day, and in the first days of the following week (23–24 March 2020), it dropped to the range of 22,000–28,000.

In the first days of the “lockdown” (25–27 March 2020), the daily number of cars passing on the street was around 20,000. It should be noted that on Sunday, 29 March 2020, street traffic was the lowest in the history of measurements, amounting to only 9282 cars passing. This proves the public’s high fear of the possibility of coronavirus infection, as well as taking into account the appeals of the Minister of Health to stay at home and limit time outside the house. However, the next week, the number of cars increased, reaching approximately 43,000 on 2 April 2020. The following days of this period are characterized by 40,000–50,000 vehicles per day. The median for this period is 44,153 per day—it is slightly lower than in period 1, “pre-pandemic,” because in the last weeks of “lockdown,” traffic began to approach the days at the beginning of the pandemic.

In the 3rd period, some of the restrictions began to be lifted. It was the period of the so-called return to normality, which the Polish government has divided into four stages. Due to this, traffic began to increase, exceeding 50,000 units per day. In the 4th period, there is another increase in the number of cars. On the last day of this period (15 May 2020), 62,000 vehicles were exceeded. The 5th period is the shortest, lasting only 1 week, during which the number of passing cars continues to increase to 65,500 units per day.

The 6th period is one of the busiest times. The number of cars driving on the street reached 70,000 on 10 June 2020—the day before the religious holiday of Corpus Christi. In later days, but after the long weekend associated with Corpus Christi, the number of cars fluctuates around 70,000 per day. The exception is 18 June 2020, on which a significant decrease in passing cars was recorded (41,479 units)—the explanation for this event is unknown.

Traffic is relatively high during the holidays (period 7), ranging from 64,000 to 72,000 cars per day. An exciting relationship can be noticed —in almost every week of the holidays, on Mondays, Tuesdays, and Wednesdays, the number of vehicles was 2000–3000 fewer than on Thursdays and Fridays. A similar situation persisted in the subsequent 8th period (Table 2). The number of cars passing by ranged from 64,000 to 72,000 vehicles per day. Only in the last 2 weeks of this period (13–23 October 2020) did the number of cars drop to 61,000 – 67,000 per day, which was caused by fear of an upcoming new wave of coronavirus infections. The weekly distribution of the number of cars is also similar to the distribution observed in period 7—the beginning of the week was characterized by fewer cars than the end of the week.

**Table 2:** Comparison of car traffic in particular periods of the pandemic

Period	1	2	3	4	5	6	7	8	9	10	11
1		*0.000*	0.437	0.080	*0.001*	*0.000*	*0.000*	*0.000*	*0.001*	*0.000*	*0.000*
2			*0.001*	*0.000*	*0.000*	*0.000*	*0.000*	*0.000*	*0.000*	*0.000*	*0.000*
3				*0.008*	*0.000*	*0.000*	*0.000*	*0.000*	*0.000*	*0.000*	*0.000*
4					*0.030*	*0.000*	*0.000*	*0.000*	0.088	*0.003*	*0.000*
5						0.271	0.143	0.088	0.326	0.950	0.132
6							0.562	0.347	*0.004*	0.075	0.522
7								0.726	*0.001*	*0.023*	0.953
8									*0.000*	*0.009*	0.771
9										0.166	*0.001*
10											*0.020*

LSD test. Variable: Car traffic. Marked differences are significant at* p* < 0.05000. Where: normal text, periods with similar car traffic; *italic text*, periods with different car traffic

Period 9 is the “2nd wave of coronavirus.” The number of cars is decreasing, ranging between 50,000 and 60,000 per day. Only on 4 November 2020 did the daily number of vehicles driven permanently exceed 60,000, but it did not exceed the upper limit of 65,600. The median is lower than in the period before the second wave (68,090 cars per day), amounting to 61,249 vehicles per day.

The next period is the “3rd wave of coronavirus.” There is an increase in the daily number of cars passing by, ranging from 6000 to 7000. The median is 64,988 cars per day. The weekly schedule changes significantly, as the fewest cars were driven on Fridays, which can be explained by part of the population working remotely. The “post-pandemic” period is characterized by a return to regular road traffic (Table 2). The daily number of cars oscillates around 70,000, and the median for this period is 69,027.

**Fig. 5:**
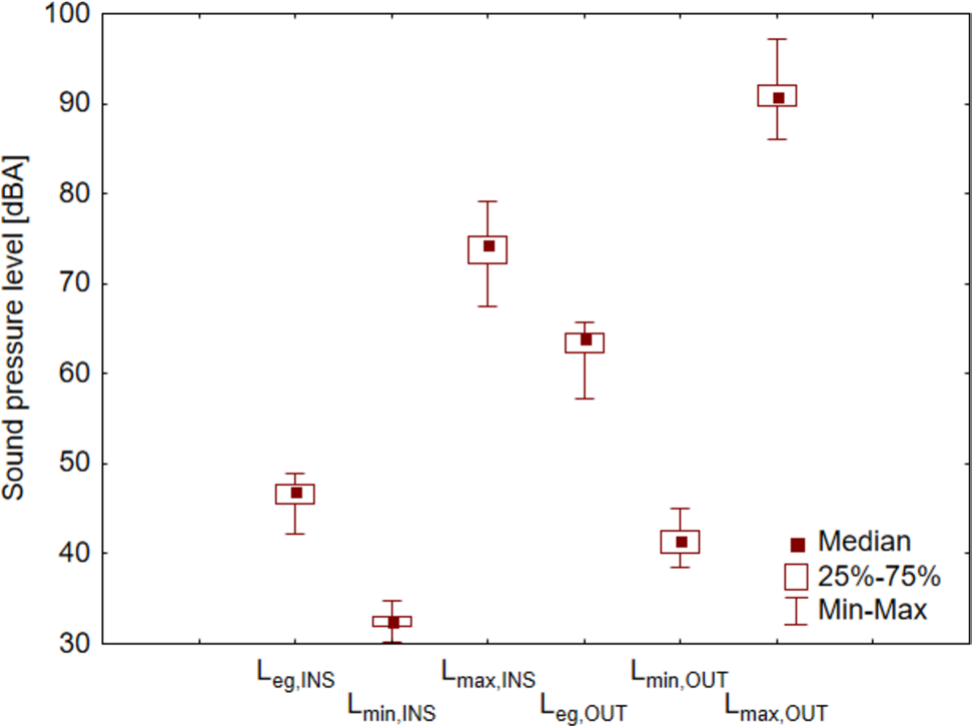
Sound pressure distribution

**Fig. 6:**
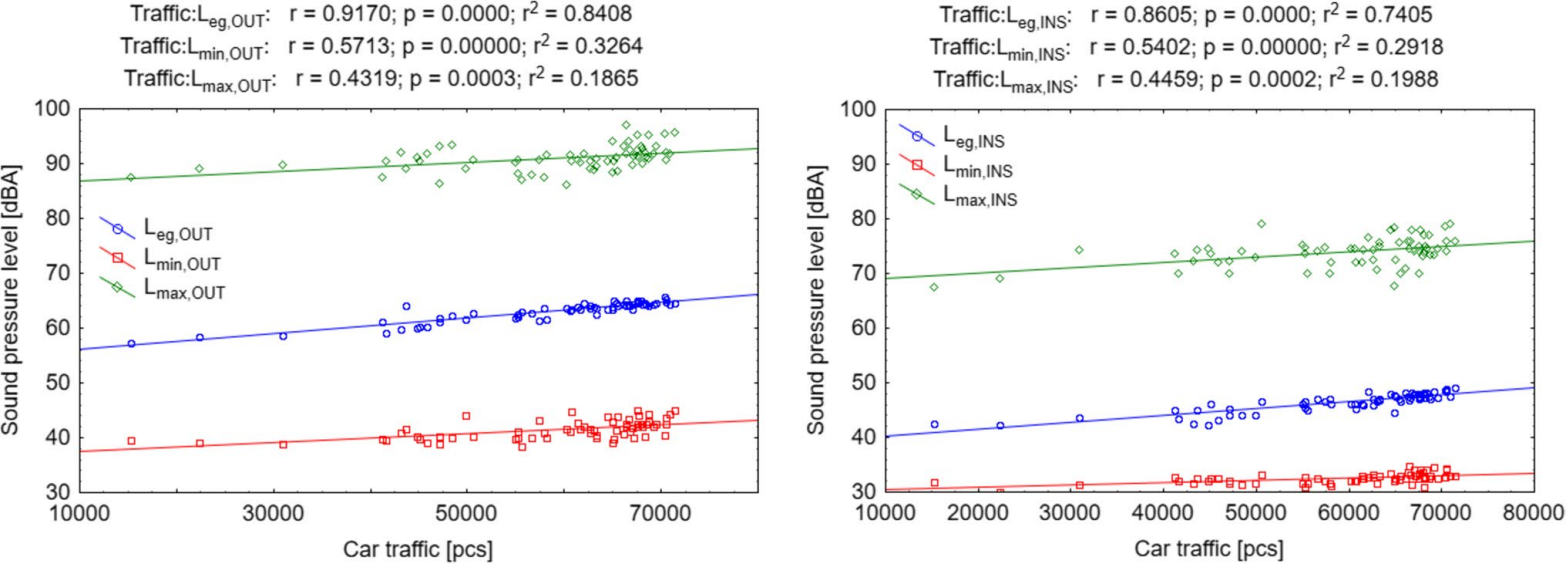
“Car traffic—sound pressure level” correlation charts outside the building (left) and inside (right)

The average daily sound pressure value (Figs. 5, 6 left) outside the *L*_eq.OUT_ building was 63.9 dBA, resulting from the prevailing street traffic (correlation coefficient, *r* = 0.92). The results confirm that vehicle traffic intensity is the main generator of road noise and significantly impacts its level (Bouzir et al. 2017; Freitas et al. 2018). The lowest average sound pressure value is *L*_min.OUT_ occurs during night hours (usually after midnight to 5:00 a.m.) but is much less correlated with street traffic (*r* = 0.57). Such a low correlation, despite the occurrence of periods without cars on the street, is caused by the influence of other sound emitters, such as passers-by or animals (barking dogs, birds singing in the morning)—so the sounds of “city” and “nature” always reached the microphone. In the case of the highest *L*_max.OUT_ noise values were caused by random events, such as the passage of emergency vehicles with signals, motorcycles, buses, or trucks (*r* = 0.43) (Rodríguez et al. 2022).

Measurements inside the building (Figs. 5, 6 right) showed similar sound pressure level correlations to traffic, although they were slightly lower. This could have been influenced by sound sources located inside the building (including noise caused by students gathering in the corridor). The sound pressure is lower than outside, in the case of average values *L*_eq_ by 15–17 dBA, absolute minimum values *L*_min_ by 8–10 dBA, and absolute maximum values *L*_max_ by 16–18 dBA.

**Fig. 7:**
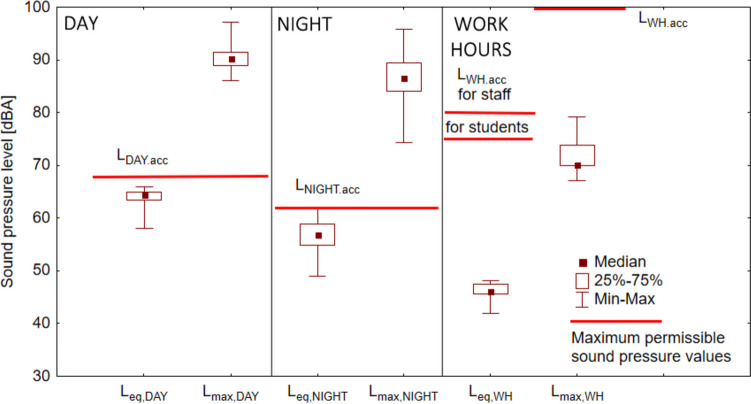
Day (*L*_eq,DAY_, *L*_max,DAY_), night (*L*_eq,NIGHT_, *L*_max,NIGHT_), and during working hours (*L*_eq,WH_, *L*_max,WH_) acoustic indicators compared with the maximum permissible values (*L*_DAY.acc_, *L*_NIGHT.acc_, *L*_WH.acc_)

According to the current Announcement (2014) (Fig. 7), during the day (from 6:00 a.m. to 10:00 p.m.), the sound pressure should not exceed 68 dBA, and at night (from 10:00 p.m. to 6:00 a.m.) 65 dBA. In the case of *L*_eq.DAY_ measurements, the values are normal. However, in the case of extreme events, *L*_max.DAY_ values are significantly exceeded. The situation is similar with night hours. The average values are within the *L*_eq.NIGHT_ standard, and the absolute maximum *L*_max.NIGHT_ exceeds the permissible limit. It should be noted that the maximum sound levels recorded during the day and at night are similar, amounting to 97.1 and 95.8 dBA, respectively, which indicates the appearance of sound emitters regardless of the time of day (passing of an emergency vehicle with a signal, a motorcycle, etc.). According to Boczkowska et al. (2014), average values during working hours should not exceed 80 dBA for university employees and 75 dBA for students. These conditions are met because the *L*_eq.WH_ sound pressure range was from 41.9 to 48.2 dBA. In the event of extreme events, *L*_max,WH_ should be below 102 dBA for an employee and 100 dBA for a student. The conditions are also met in both cases, as the highest sound level recorded was 79.2 dBA.

**Fig. 8:**
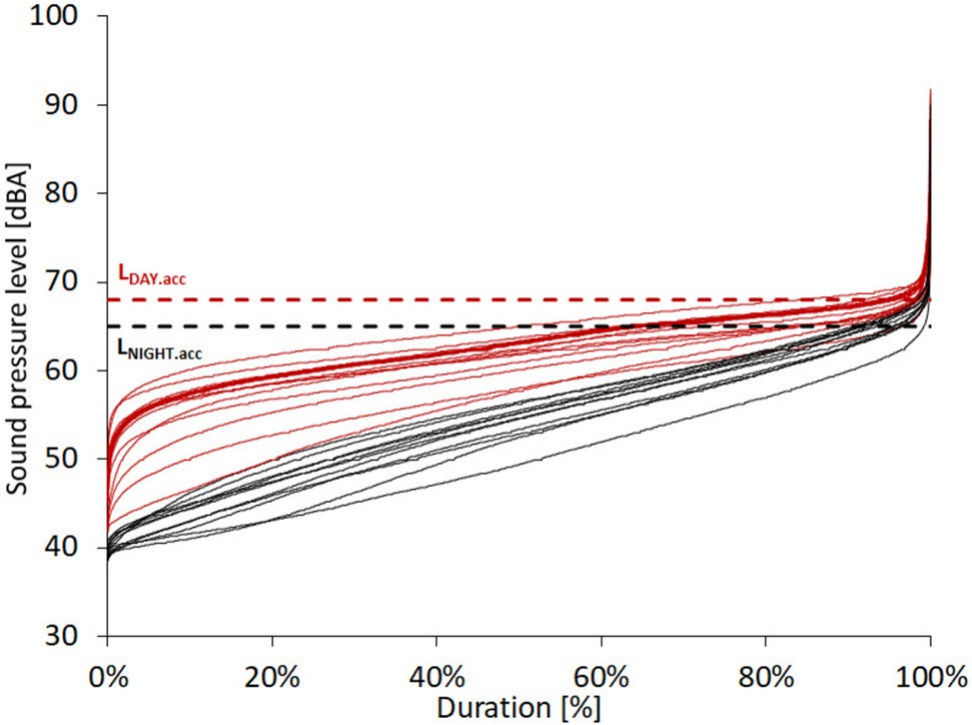
Frequency of measurements during the day and night outside the building (some example)

The duration of the noise (Fig. 8) (in this case, noise is considered to be exceeding the limit values presented in the Announcement (2014)) is relatively short-lived. It is usually associated with the passage of vehicles with high sound emissions, such as emergency cars, motorcycles, trucks, and delivery vehicles. During the day, only approximately 3.1% (45 min per day) of the time during the day exceeds the permissible level. However, the range varies from 0.7% (on Sundays and holidays) to 16.2% on days with intense traffic (10 min–3 h 53 min respectively). At night, the median time of exceeding the permissible values is higher, amounting to 7.0%, 1 h 41 min (range 0.6–11.4%, 9 min–2 h 44 min). The highest momentary recorded sound pressure value *L*_max.OUT_ from all measurement days was 97.1 dBA—by OSHA (2022) recommendations, a person may be exposed to such high noise for 3 h.

**Fig. 9:**
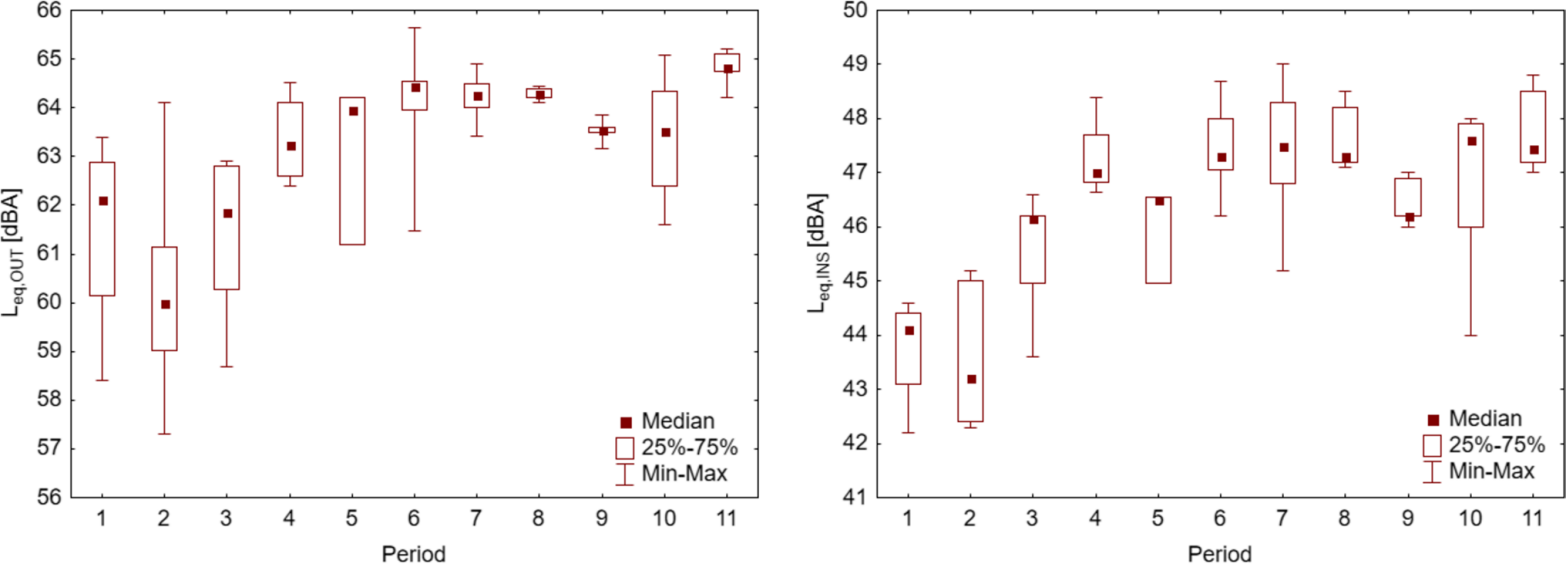
Sound pressure level outside the building (left) and inside the building (right) during individual restriction periods

Analyzing the noise (Fig. 9) caused by road traffic in particular periods of the pandemic, as previously stated, it strongly depends on road traffic on Mickiewicza Street in Krakow (Fig. 6).

Outdoors the building (Fig. 9, left), the noise during the initial period of the coronavirus pandemic (periods 1–3) was comparable (Table 3). In the following period 3, the sound pressure level increases, which is influenced by the decision of the Minister of Health to partially lift restrictions partially, thus increasing car traffic. According to Table 3, the 5th “3rd stage” period is similar to the noise occurring during the 1st “pre-lockdown” period and the 3rd “1st stage” period. Please remember that the 5th period is short, lasting only 8 days. Hence, the data measured then may be unreliable. The remaining periods (4–11) are comparable. Despite this, the noise outside the building decreased slightly in the 9th “2nd wave.”

**Table 3:** Comparison of sound pressure (*L*_eq.OUT_) outside the building in particular periods of the pandemic

Period	1	2	3	4	5	6	7	8	9	10	11
1		0.123	0.816	*0.049*	0.111	*0.003*	*0.002*	*0.002*	*0.019*	*0.012*	*0.000*
2			0.164	*0.000*	*0.002*	*0.000*	*0.000*	*0.000*	*0.000*	*0.000*	*0.000*
3				*0.022*	0.061	*0.001*	*0.000*	*0.000*	*0.006*	*0.003*	*0.000*
4					0.810	0.476	0.298	0.270	0.835	0.833	0.087
5						0.360	0.227	0.207	0.654	0.642	0.069
6							0.654	0.587	0.576	0.521	0.193
7								0.914	0.351	0.296	0.405
8									0.316	0.266	0.485
9										0.988	0.092
10											0.063

LSD test; variable: *L*_eg,OUT_. Marked differences are significant at *p* < 0.05000. Where: see Table 2

Inside the building (Fig. 9, right), the distribution of sound pressure values is similar to the noise outside (*r* = 0.84). The sound pressure levels in the 1st and 2nd periods are similar (Table 4) despite the most stringent restrictions introduced in period 2, such as the prohibition of movement freedom, the closure of educational institutions, and the introduction of online learning. In subsequent periods, restrictions began to be lifted, and a 4th stage “return to normality plan” was introduced. Even though it was only in the 6th period (“4th stage”) that stationary education was partially restored (including for students taking classes that cannot be held “online” and for students in their final years of studies), already in stage 3, an increase in noise can be seen in the building. The reason for this is greater traffic outside the building, which was associated with easing restrictions. The 6th period (“4th stage”) was before the holidays; for students, it was a period of final exams. As a result of the partial opening of educational institutions, the return of teachers to schools, and students staying in university buildings, there is a further increase in noise inside the facility. In some way, workers and students also contributed to the increase in noise, but with heavy car traffic, this increase is statistically insignificant. During the holidays (period 7), a similar amount of noise was detected as in the previous period. A greater dispersion of values can be noticed due to the specificity of the university’s work. In July, tests, sessions, and exams take place—so it is still a period of work and study for students—so the sound pressure is higher; and in August, when students are on holiday, the sound pressure level is lower. In the 8th period, the school year (1 September 2020) and the academic year (1 October 2020) began. Resit examination sessions were held in September, and full-time classes were held in October. Therefore, the sound pressure level was relatively high and comparable to the “post-pandemic” period (Table 4). Only in the 9th period, “2nd wave,” when educational institutions were closed again, did the sound pressure level decrease. In the next period, the 10th “3rd wave,” the sound pressure level increased even though educational institutions continued to provide online teaching.

**Table 4:** Comparison of sound pressure level (*L*_eq.INS_) inside the building in particular periods of the pandemic

Period	1	2	3	4	5	6	7	8	9	10	11
1		0.633	*0.013*	*0.000*	*0.006*	*0.000*	*0.000*	*0.000*	*0.000*	*0.000*	*0.000*
2			*0.001*	*0.000*	*0.001*	*0.000*	*0.000*	*0.000*	*0.000*	*0.000*	*0.000*
3				*0.013*	0.509	*0.001*	*0.002*	*0.001*	0.146	*0.020*	*0.001*
4					0.111	0.775	0.779	0.611	0.205	0.522	0.478
5						*0.039*	*0.045*	*0.031*	0.566	0.208	*0.020*
6							0.994	0.766	0.063	0.240	0.586
7								0.783	0.076	0.268	0.611
8									0.049	0.179	0.822
9										0.407	*0.030*
10											0.113

LSD test. Variable: *L*_eg,INS_. Marked differences are significant at* p* < 0.05000. Where: see Table 2

Another issue was the assessment of meteorological conditions concerning potential noise pollution. The values of meteorological parameters were variable during the day. For example, the lowest temperature occurred just before sunrise and the highest between 3:00 p.m. and 6:00 p.m., depending on the season. Daily differences could reach up to 20 °C—especially in spring. Humidity was also highly variable, being driest during the day and most humid at night. The correlation between momentary humidity and air temperature values during the day is high, oscillating around − 0.97. Even though the wind speed depended on barometric conditions and the atmospheric pressure gradient, daily variations were often observed (lower at night, higher during the day). Therefore, the correlation of instantaneous values during the day oscillated around − 0.73 for humidity and 0.73 for temperature.

To determine the impact of metrological conditions on sound pressure, average daily temperature, humidity, and wind speed were taken. In the case of precipitation, its daily sum was analyzed. Table 5 shows the correlation coefficients between meteorological and acoustic parameters. Low correlation coefficients indicate a small impact of meteorological conditions on the noise level, which was also confirmed in their studies (Chowdhury et al. 2015; 2016; Munir et al. 2021).

**Table 5:** Correlation of different weather conditions and acoustic parameters*

Acoustic parameters	Mean velocity wind	Mean humidity	Mean air temperature	Sum participation
*L*_eq_	− 0.177	0.442	0.303	0.141
*L*_eq.DAY_	− 0.044	0.331	0.040	0.172
*L*_eq.NIGHT_	− 0.161	0.453	0.622	0.025
*L*_eq.WH_	0.288	0.304	− 0.364	− 0.051

^*^Correlation is significant at the 0.05 level

The behavior of society during the COVID-19 pandemic was more dependent on the restrictions introduced by the Minister of Health and the fear of infection with the SARS-CoV-2 coronavirus than on the development of the pandemic. The low correlation between traffic intensity and infections/deaths demonstrates this (Table 6).

**Table 6:** Correlation of social mobility and the development of the COVID-19 pandemic*

	Infections per week	Deaths per week
Number of vehicles passing on the street during the week	0.119	0.176
Infections per week		0.972

^*^Correlation is significant at the 0.05 level

Despite the small number of infections and deaths during the lockdown (period 2), society’s mobility was limited to a minimum. Also, society’s fear of a new, unknown virus caused people to comply with the introduced restrictions. High penalties for failing to comply with the restrictions were undoubtedly another reason for compliance with the introduced orders and prohibitions.

In the following periods (nos. 3–7), restrictions were lifted, increasing mobility and street traffic.

It was not until the second wave of the pandemic (period 9) that restrictions began to be reintroduced. Despite the large numbers of infected and deceased people, the mobility of society decreased slightly. This can be especially noticeable during the third wave of the pandemic (period 10), even though more restrictive restrictions were introduced than during the second wave (period 9). During the third wave of the pandemic, part of society had already been vaccinated against the SARS-CoV-2 coronavirus, so the fear of infection was lower than earlier. The vaccination program began on 27 December 2020, and was only for medics. On 15 January 2021, the vaccination program was extended to seniors aged 80 + and 10 days later to people aged 70 + . In mid-February 2021, the program was expanded again to teachers. At the beginning of May, any adult person could join the program (Ministry of Health 2024). Likely, the vaccination program, the reluctance of society to comply with restrictions, fatigue with the inconveniences associated with the application of restrictions, and the calming down of the pandemic between the first and second waves caused society to become accustomed to the threat and stop fearing infection.

Politicians also used the COVID-19 pandemic for propaganda purposes. The presidential elections were held in June/July 2020. During the campaign, politicians made many statements encouraging citizens to participate in the elections in person and support their candidate. The persuasions were accompanied by slogans about the lack of the virus in society, about defeating the virus, about not being afraid of the virus, and about the retreat of the virus. The behavior of politicians also aroused extreme emotions in society. During the lockdown, politicians from the ruling team often did not comply with the restrictions, which resulted in later moral scandals. During this period (nos. 6–7) society was firmly convinced that the coronavirus “… is in retreat.” “There is no need to be afraid of it now,” said the Prime Minister during a political rally in Tomaszów Mazowiecki on 2 July 2020. Moreover, despite the experts’ appeals to maintain the sanitary regime, because the pandemic has not ended yet and will intensify in the fall, populist slogans won by lulling citizens’ vigilance and reducing their fear of the coronavirus.

## Discussion

Around Mickiewicza Street, one of the city’s main thoroughfares (the so-called second ring road) connects the city’s northern districts with the southern ones, the main noise emitter is street traffic. During the COVID-19 pandemic, traffic volumes have decreased, resulting in less noise in surrounding areas. The correlation between traffic and noise pollution is *r* = 0.92 for *L*_eq,OUT_. Our observations are the same as those measured by Alsina-Pagès et al. (2021), Vida Manzano et al. (2021), and Rodríguez et al. (2022), who also showed a reduction in noise pollution due to reduced street traffic. Noise pollution as a result of the introduction of restrictions related to the COVID-19 pandemic decreases on average by 5.0–10.0 dBA in Lorient, 6.6 dBA in London, 6.0 dBA in Milan, and 1.4–4.7 dBA in Buenos Aires (Alsina-Pagès et al. 2020; Aletta et al. 2020; Alías-Pujol et al. 2024; Aumond et al. 2022; Said et al. 2020; Zambon et al. 2021). Our measurements show that noise during the pandemic (period 2 “lockdown”) decreased by 4.83 dBA, which was a value more similar to the studies performed in Rio de Janeiro (3.0–5.0 dBA), Rome (5.2 dBA), Madrid (3.9–7.4 dBA), in Paris (4.5 dBA), and Stockholm (4.0 dBA) (Abdmouleh and Dahech 2024; Alsina-Pagès et al. 2020; Asensio et al. 2020; Gevú et al. 2021; Rumpler et al. 2020). Gevú et al. (2021) state that the decrease in sound pressure was slight compared to limited vehicle traffic, which decreased by up to 50%. Our measurements confirm this observation that despite a significant decrease in traffic (36% in period 2 compared to period 11), noise pollution in the area was reduced by only 4.83 dBA (only 7.45% compared to period 11). The noise reduction seems small; however, at a value of 4.83 dBA, the acoustic power decreases by about 3.5 times, so human perception can significantly feel this change.

The data presented above are average values (*L*_eq,OUT_). Analyzing the minimum data (*L*_min,OUT_), they depend on a lesser extent on the traffic intensity (*r* = 0.57). Such a low correlation, despite the occurrence of periods without cars on the street, is caused by the influence of other sound emitters, such as passers-by or animals (barking dogs, birds singing in the morning)—so the sounds of “city” and “nature” always reached the microphone. In the case of the highest *L*_max.OUT_ noise values were caused by random events, such as the passage of emergency vehicles with signals, motorcycles, buses, or trucks (*r* = 0.43). Our observations were also confirmed by Rodríguez et al. (2022).

The noise caused by the engines and drives of trucks is very bothersome (Jensen 1973; Sburlati et al. 2019). In the case of the analyzed road, cars over 3.5 tons can drive on the road, but due to the more convenient passage via the IV ring road, which has the status of a motorway, their number on Mickiewicza Street is negligible. According to Gierasimiuk and Motylewicz (2014), the sound pressure is influenced by the number and speed of driving cars. On the analyzed road section, the speed limit is 50 km/h, but during rush hours, the speed of moving vehicles is much lower and is interrupted by stops at intersections or traffic jams. The morning peak lasts from 6:30 to 8:00 a.m., and the afternoon peak from 3:00 to 5:00 p.m. (sometimes until 6:00 p.m.)—then the driving takes place intermittently. However, despite observations (Bazaras et al. 2010), this does not cause significant changes in the noise level in the environment. During the morning peak hours (weekly), the highest noise is observed, which is, on average, 3 dBA higher than the afternoon peak, which in turn is only 1 dBA higher on average than the period around noon (8:00 a.m.–3:00 p.m.). The above values increase sound pressure by 2 × and 1.25 × , respectively (Poniatowski 2022). Gardziejczyk (2018) and Bohatkiewicz et al. (2014) claim that increasing the speed of vehicles increases the noise caused by their movement. This thesis is actual, but only in the case of individual vehicles or a representative group of cars. When there is a low density of vehicles on the road outside rush hours, their speed increases, sometimes exceeding the speed limit, but the noise decreases. Therefore, the thesis that road noise depends mainly on the intensity of traffic is confirmed (Kumar et al. 2014).

Asensio et al. (2020) found that the most significant decreases in noise pollution were recorded in the area of large thoroughfares. They noticed that the temporal distribution of outdoor activities had changed. During the COVID-19 pandemic, increased traffic started and ended earlier in the day. In our research, during various periods of pandemic restrictions, street traffic patterns were always similar on individual days of the week. On working days, the morning peak lasts from 6:30 a.m. to 8:00 a.m., and the afternoon peak from 3:00 p.m. to 5:00 p.m. (sometimes until 6:00 p.m.). On Saturdays, the peak time is from 11:00 a.m. to 2:00 p.m. On Sundays, from 6:00 p.m. to 7:30 p.m. In particular periods of restrictions, only the traffic intensity varied. During the pandemic (especially period 2 “lockdown,” but also in periods 1 and 3), noise dynamics (the range between the maximum and minimum sound pressure values) increased, which was also observed in their research by Asensio et al. (2020). The increase in noise dynamics results from decreased sound pressure during off-peak hours, when fewer cars pass by, resulting in lower values from the lower measurement ranges. In turn, the maximum values remained at a similar level as outside the pandemic period because random events, such as the passage of emergency vehicles with signals, motorcycles, buses, or trucks, caused them.

During the COVID-19 pandemic, soundscapes changed: locations previously dominated by traffic noise became more pleasant. On the other hand, after the end of the periods of restrictions caused by the COVID-19 pandemic, places with reduced noise pollution returned to the original sound level, which, although perhaps even within standards, could be bothersome (Mitchell et al. 2021).

## Conclusions

Sound pressure research and measurements provide information on the exposure scale of people living and working near roads to noise. Medicine informs about the effects of human exposure to long-term and bothersome noise. Our measurements did not show that the noise persisted for a long time. Typically, if the maximum permissible sound pressure values are exceeded, it is only for short periods and should not adversely affect human health. However, this does not mean that attempts to reduce noise pollution should be abandoned. Unfortunately, Polish legislation goes in the opposite direction — it increases the maximum permissible sound pressure values that may occur near emitters. However, the awareness of citizens and the actions of local governments and road managers resulted in introducing more and more effective measures to reduce noise nuisance. Such activities include, among others, the introduction of a strategy for protection against noise pollution, analysis of acoustic maps, and in cases of noise originating mainly from road traffic, limiting the speed of moving vehicles, entry ban for trucks and particularly noisy vehicles, maximizing traffic continuity, the so-called green line, drivers’ awareness and driving culture, ban on honking at night, construction of a soundproofing surface on the road, and construction of noise barriers.

There is no current noise pollution monitoring network in Poland. The statistical data analysis allowed us to create a regression formula, which, based on the number of moving vehicles, we can assess noise pollution in a 15-min interval.

The COVID-19 pandemic, and especially the restrictions introduced by the Polish Ministry of Health, resulted in reduced mobility (especially in the first phase of the pandemic), which translated into reduced road noise pollution. Based on our research and analyses, we can also demonstrate three stages that show society’s approach to the COVID-19 pandemic:

1. **Fear of the COVID-19 pandemic.** This stage includes periods 1, 2, and 3. The street noise decreased (to 62.11 dBA outside the building), caused by reduced street traffic (43,614 cars per week). The first restrictions were then introduced, and people usually complied with them for fear of being infected with the new, not fully understood coronavirus.
2. **Relaxation after restrictions.** This is the second stage of lifting restrictions (4th period). Fifty days have passed since the introduction of the lockdown (period 2), and 14 days have passed since the first restrictions were lifted. At this stage, shopping malls, cultural centers, and sports fields were opened, enabling outdoor sports. Society was tired of weeks of restrictions, so it started using the infrastructure. Part of the population returned to stationary work. The number of cars on the streets increased to 55,263 per week, and the noise level increased to 63.25 dBA. This was a period in which society began to get used to the new pandemic situation in the country, especially since the number of infections was still not high.
3. **Getting used to the COVID-19 pandemic.** This stage includes periods 5–10. Despite the reintroduction of restrictions, people complied with them with moderate results. People have become accustomed to the threat, despite the second and third waves of the pandemic occurring during this period, in which the number of infections and deaths was several dozen times higher than during the first wave. Road traffic returned to its original level and, with minor exceptions (2nd wave: 60,232 cars per week, 63.55 dBA, which was caused by the 2nd lockdown), remained at non-pandemic levels (above 65,000 vehicles per week, and noise fluctuated around 64.00 dBA).

## Data Availability

The datasets used and/or analyzed during the current study are available on reasonable request.
